# Energy Conservation and the Promotion of *Legionella pneumophila* Growth: The Probable Role of Heat Exchangers in a Nosocomial Outbreak

**DOI:** 10.1017/ice.2016.205

**Published:** 2016-09-19

**Authors:** Emilie Bédard, Simon Lévesque, Philippe Martin, Linda Pinsonneault, Kiran Paranjape, Cindy Lalancette, Charles-Éric Dolcé, Manuela Villion, Louis Valiquette, Sébastien P. Faucher, Michèle Prévost

**Affiliations:** 1 Department of Civil Engineering, Polytechnique Montréal, Montréal, Canada; 2 Laboratoire de Santé Publique du Québec/ Institut National de Santé Publique du Québec, Sainte-Anne-de-Bellevue, Canada; 3 Department of Microbiology and Infectious diseases, Faculty of Medicine and Health Sciences, Université de Sherbrooke, Sherbrooke, Canada; 4 Department of Community Health Sciences, Faculty of Medicine and Health Sciences, Université de Sherbrooke, Sherbrooke, Canada; 5 Department of Natural Resource Sciences, Faculty of Agricultural and Environmental Sciences, McGill University, Sainte-Anne-de-Bellevue, Canada; 6 Centre d’expertise en analyse environnementale du Québec, Québec, Canada

## Abstract

**OBJECTIVE:**

To determine the source of a *Legionella pneumophila* serogroup 5 nosocomial outbreak and the role of the heat exchanger installed on the hot water system within the previous year.

**SETTING:**

A 400-bed tertiary care university hospital in Sherbrooke, Canada.

**METHODS:**

Hot water samples were collected and cultured for *L. pneumophila* from 25 taps (baths and sinks) within wing A and 9 taps in wing B. Biofilm (5) and 2 L water samples (3) were collected within the heat exchangers for *L. pneumophila* culture and detection of protists. Sequence-based typing was performed on strain DNA extracts and pulsed-field gel electrophoresis patterns were analyzed.

**RESULTS:**

Following 2 cases of hospital-acquired legionellosis, the hot water system investigation revealed a large proportion of *L. pneumophila* serogroup 5 positive taps (22/25 in wing A and 5/9 in wing B). High positivity was also detected in the heat exchanger of wing A in water samples (3/3) and swabs from the heat exchanger (4/5). The outbreak genotyping investigation identified the hot water system as the source of infections. Genotyping results revealed that all isolated environmental strains harbored the same related pulsed-field gel electrophoresis pattern and sequence-based type.

**CONCLUSIONS:**

Two cases of hospital-acquired legionellosis occurred in the year following the installation of a heat exchanger to preheat hospital hot water. No cases were reported previously, although the same *L. pneumophila* strain was isolated from the hot water system in 1995. The heat exchanger promoted *L. pneumophila* growth and may have contributed to confirmed clinical cases.

*Infect. Control Hosp. Epidemiol.* 2016;1475–1480

Each year, hospital-acquired legionellosis cases result in prolonged hospitalization with elevated mortality rates.^1^ These cases are predominantly associated with *Legionella pneumophila* serogroup 1 (sg1) strains present in the hospital hot water systems.^2^ ^–^ ^4^ A few outbreaks and isolated cases have been related to *L. pneumophila* (*Lp*) serogroup 5 (sg5).^5^ ^–^ ^7^ Several factors can contribute to *Legionella* growth and persistence within hospital water systems: temperature, stagnation, biofilm, material, disinfectant, and water quality.^8^ ^–^ ^12^ Key measures to control *Lp* in hot water systems are to maintain elevated water temperatures throughout the system and to minimize stagnation through optimal water circulation.^13^ ^–^ ^16^ Although an infectious dose has not been determined, several countries have established action levels between 1,000 and 10,000 colony-forming units (CFU)/L, and a concentration higher than 10,000 CFU/L requires immediate corrective actions.^13^ ^,^ ^15^ ^,^ ^17^ ^–^ ^19^

At the same time, healthcare facilities are encouraged to implement energy and water conservation devices to meet accreditation requirements, such as Leadership in Energy and Environmental Design certification. The use of waste heat recovery systems to preheat hospital hot water prior to the water heater is an option offered to hospitals to increase energy efficiency. However, operational practices and ideal growth conditions associated with these devices can promote the development and persistence of *Lp* unless a thorough risk assessment is performed. In this study, we report a nosocomial outbreak of *Lp* sg5 in the year following the installation of 2 heat exchangers in 2 distinct hospital wings as part of an energy conservation upgrade. The objectives of our study were to determine the source of the outbreak and to understand the role of the heat recovery systems.

## METHODS

Two nosocomial cases of *Lp* were reported in August 2014 within a wing (wing A) of a 400-bed tertiary care university hospital in Sherbrooke, Canada. Clinical samples were cultured on buffered charcoal-yeast extract (BCYE) medium, and isolates were sent to the Laboratoire de Santé Publique du Québec for identification and serogroup confirmation as described previously.^20^ Following the reported cases, 250 mL of first flush hot water were collected from 25 taps (baths and sinks) within wing A (300 beds) and from 9 taps within wing B (100 beds, supplied by a separate hot water system). The heat exchangers from wings A and B were investigated in June and July 2015, respectively. Biofilm samples and water (2 L) were collected from 3 locations: at the water inflow pipe, inside the heat exchanger, and at the water outflow pipe. Two additional biofilm samples were recovered within the heat exchanger. Environmental samples were cultured according to the Association Française de Normalisation NF T90-431 method^21^ with the addition of a 1-mL filtration. Briefly, different volumes were filtered through sterile 47 mm diameter 0.45 µm mixed ester cellulose membranes (Millipore) and an untreated sample volume of 0.2 mL was plated on glycine-vancomycin-polymyxin-cycloheximide selective agar (Biokar Diagnostics) and incubated at 36°C for 10 days. Before plating, acid treatment was applied to filtered samples (pH, 2; 5 min). Typical colonies that developed after 4 to 10 days were subcultured on confirmation plates (BCYE agar without and with cysteine) for 2 to 4 days, at 36°C. Resulting colonies that developed on BCYE agar, but not on BCYE without cysteine, were considered as *Legionella* spp. The *Legionella* latex test (DR0800; Oxoid) was used for *Lp* confirmation. The calculated detection limit for the culture method was 10 CFU/L for both *Legionella* spp. and *Lp*. Pulsed-field gel electrophoresis (PFGE) and sequence-based typing (SBT) were performed as described previously.^20^ In some cases, nested-SBT protocol was performed on DNA extracts obtained from water sampling.^22^ PFGE patterns were analyzed according to the criteria of Tenover et al^23^ and with BioNumerics software, version 6.5 (Applied Maths), by the unweighted pair-group method with arithmetic average clustering method using the Dice coefficient with both position tolerance and optimization of 1%. Biofilm and water samples from the heat exchanger were analyzed for the presence of protists through direct microscopy and 18s polymerase chain reaction amplification using the following primers: Euk1A (5′-CTGGTTGATCCTGCCAG-3′); Euk516r (5′-ACCAGACTTGCCCTCC-3′).

Statistical analysis (*Z*-test and Kruskal-Wallis) was performed with Statistica, version 10 (Dell), to compare the percent positivity and level of *Lp* in wing A and wing B. The significance level was set at *P*=.05.

## RESULTS

Nosocomial legionellosis was diagnosed in 2 patients hospitalized in wing A (August 2014), and clinical specimens were positive for *Lp* sg5. The first case occurred in the oncology ward, on the seventh floor of wing A. The patient was admitted on July 12 with acute myeloid leukemia. The first signs of pneumonia appeared on August 1. A sample was collected through bronchoalveolar lavage and the presence of *Lp* sg 2–14 was confirmed. The second case occurred in a patient admitted on July 25 to the medical intensive care unit (ninth floor, wing A) for a third-degree atrioventricular block. His main comorbidity was a chronic obstructive pulmonary disease for which he took prednisone. He had a permanent pacemaker placement on July 26 and was transferred to the surgical ward (eighth floor, wing A), where he stayed from July 27 to August 5. Significant coronary artery disease was discovered and he underwent coronary artery bypass graft surgery on August 5. Following this intervention, he was transferred to the surgical intensive care unit (third floor, wing B), where he remained for the rest of his stay. The first signs of pneumonia appeared on August 8 and clinical samples were recovered through bronchoscopy. The presence of *Lp* sg 2–14 was also confirmed. Provided the incubation time of 2 to 10 days, the patient was possibly exposed during the 3-day stay in wing B (intensive care unit) but most probably during the 10-day stay in the surgical ward in wing A.

Environmental investigation from the hot water systems revealed a large proportion of *Lp* sg5 positive taps with high levels of contamination (88% in wing A and 56% in wing B; Table 1), whereas there was no *Lp* detected in the cold water feeding into the hospital.^9^ The percentage of positive taps and the level of contamination by *Lp* were significantly higher in wing A compared with wing B. A copper-silver ionization treatment was present on both hot water systems at the time of the outbreak. Disinfection by heat shock (≥60°C for ≥7 minutes at each tap) was conducted as previously described^9^ in wing A (August 2014) and in wing B (September 2014). The disinfection was followed by the implementation of a higher temperature set point at the water heater outlet (≥60°C) in both wings. Following temperature corrective measures, no cases of legionellosis were reported despite written directives asking hospital physicians to obtain *Legionella* cultures of respiratory specimens for all cases of nosocomial pneumonia. No *Lp* were detected in any of the water and biofilm heat exchanger samples from wing B (including the feed and outflow water samples), whereas high positivity for *Lp* sg5 was detected in wing A in water samples (3/3) and swabs from the inner surface of the heat exchanger (4/5). A gradient of *Lp* was observed in water samples from wing A heat exchanger: 510 CFU/L in the feed water, 5,000 CFU/L in the heat exchanger water, and 88,000 CFU/L in the outflow water, prior to the water heater. Protists were not readily observed by microscopy in any of the collected samples. Polymerase chain reaction also failed to detect protists in the biofilm swabs and in water samples. The heat exchangers were stopped at the time of the sampling and have not been back in service since.^9^

**TABLE 1:** *Legionella pneumophila* Positivity and Levels Measured in Hot Water Sampled From Taps in Wing A and Wing B at the Time of the Outbreak (August 2014)

***L. pneumophila*** **positivity**	**Wing A**	**Wing B**
Number of sampled taps	25	9
Number of *L. pneumophila* positive taps	22	5
Mean *L. pneumophila,* CFU/L	27,200	1,700
Standard deviation, CFU/L	18,921	1,857
Median *L. pneumophila,* CFU/L	18,000	1,000
Maximum *L. pneumophila* level, CFU/L	80,000	5,000

NOTE. CFU, colony-forming units.

**FIGURE 1:**
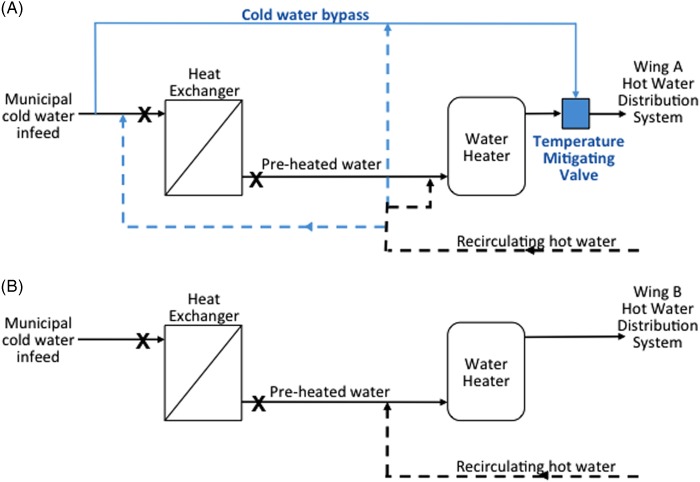
Hot water production unit flow diagram for wing A (A) and wing B (B). Differences between the 2 systems are highlighted in a different color; the letter X indicates sampling locations before and after the heat exchangers.

The hospital hot water was preheated with residual energy from the building heating system loop with single-pass heat exchangers (34 plates in wing A and 21 plates in wing B). The available surfaces were estimated at 15 m^2^ (wing A) and 5.5 m^2^ (wing B), with water volumes of 11.4 L and 3.8 L, respectively, resulting in a very high surface-to-volume ratio of 14 cm^−1^. Temperatures within the heat exchangers ranged from 9°C to 46°C, and prolonged stagnation was observed during the night, resulting in no flow for 48% and 51% of the time in wing A and wing B, respectively. The average flow rates during daily operation were estimated to be 18 L/min in wing A and 43.8 L/min in wing B, well below the maximum designed flow rate of 230 L/min. Figure 1 illustrates the flow diagram of the heat exchangers for both wings. The hot water temperature feeding into both distribution systems was less than 55°C before the outbreak.

**FIGURE 2:**
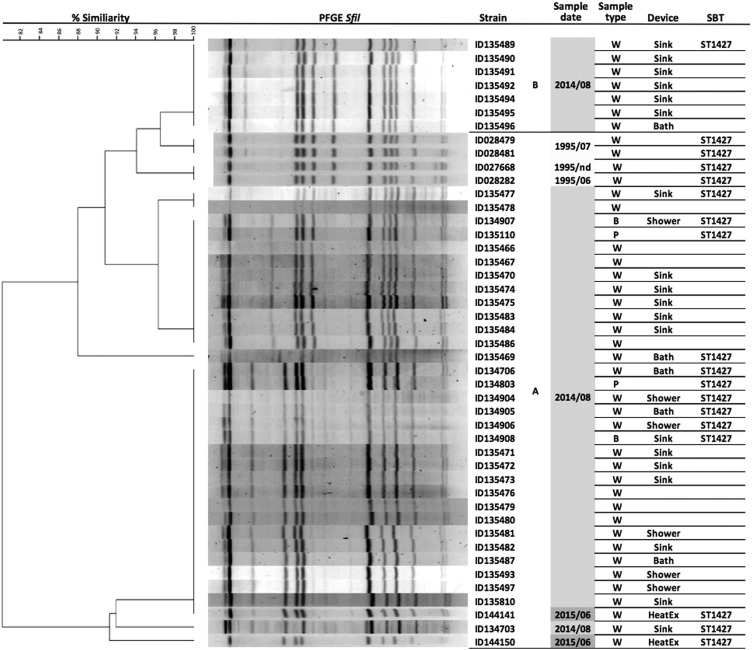
Pulsed-field gel electrophoresis (PFGE) patterns and sequence-based typing (SBT) types of *Legionella pneumophila* serogroup 5 isolated from clinical and environmental samples.

In total, 34 clinical and environmental isolates (wing A), 7 environmental isolates (wing B), and 4 environmental isolates collected in 1995 (wing A) were typed by PFGE and SBT (Figure 2). Environmental isolates dating from 1995 were collected from the hot water system in wing A as part of a previous case investigation, where the source of infection was found to be unrelated to this hospital. Genotyping results revealed that all isolated environmental strains (1995 and 2014) harbored the same related PFGE pattern as the outbreak strain (Figure 2). All typed isolates were also from the same SBT type (ST-1427). Partial SBT profiles were obtained from 12 additional hot water sample DNA extracts using the nested-SBT protocol (Table 2), collected a year after the outbreak.^9^ The obtained alleles were similar to ST-1427 for 11 of the 12 DNA extracts, suggesting the presence of the same strain in those water samples.

**TABLE 2:** Sequence-Based Typing (SBT) Results From 12 Hot Water Sample DNA Extracts Collected in Wing A and Wing B in July 2015^9^

		**SBT allele sequence number**							
**Sample no.**	**Location**	***flaA***	***pilE***	***asd***	***mip***	***mompS***	***proA***	***neuA*** **h**	**ST**
ID145265	Wing A (Tap)	3	12	1	6	14	9	F	1427-*like*
ID145266	Wing A (Tap)	3	12	1	6	14	9	F	1427-*like*
ID145267	Wing A (Tap)	3	F	F	6	14	9	F	1427-*like*
ID145268	Wing A (Tap)	3	12	1	6	14	F	F	1427-*like*
ID145269	Wing A (Tap)	3	12	1	6	14	F	F	1427-*like*
ID145270	Wing A (Tap)	3	12	NEW	6	F	NEW	F	New profile
ID145329	Wing B (Tap)	3	12	1	6	14	9	F	1427-*like*
ID145330	Wing B (Recirc)	3	12	1	6	14	9	F	1427-*like*
ID145331	Wing B (Tap)	3	12	1	6	14	9	F	1427-*like*
ID145332	Wing B (Tap)	3	12	1	6	14	9	F	1427-*like*
ID145333	Wing A (Heat exchanger water infeed)	3	12	1	6	14	9	F	1427-*like*
ID145334	Wing A (Heat exchanger water infeed)	3	12	1	6	14	9	F	1427-*like*
Outbreak strain		3	12	1	6	14	9	220	1427

NOTE. F, polymerase chain reaction amplification failed; NEW, allele number pending; ST, sequence type.

## DISCUSSION

The environmental investigation indicated the hot water system as the most probable source of the outbreak. Although *Lp* was detected in both systems investigated, the level of contamination was significantly higher in wing A. Despite the observed reduction in hot water contamination levels after the heat shock disinfection,^9^ *Lp* was still detected by culture in more than 45% of faucets and in the recirculation water for both wings in February 2015, 6 months after the implementation of corrective measures. The high level of *Lp* outbreak strain detected in all water samples from the heat exchanger in wing A at the time of sampling suggests its potential colonization and role at the time of the outbreak. Although no protists could be isolated, their presence in heat exchangers should also be monitored in light of the manufacturer’s warning of biofouling risk due to organisms such as protozoa, a natural reservoir for *Lp*.^24^

The relatedness of the environmental strains (n=4 in 1995; n=39 in 2014–2015) and patient strains (n=2) confirms a system-wide contamination with the established *Lp* sg5 strain and suggests its persistence over a period of 20 years in the hot water system. The heat exchanger in wing A promoted the *Lp* sg5 genotype present in the system, whereas no colonization of the wing B heat exchanger was identified. Detailed investigation of the flow diagrams and onsite validation showed important differences between the design and operation of the 2 hot water systems, including the heat exchanger configuration (Fig. 1). The heat exchanger from wing A was fed by a combination of cold makeup water and recirculated hot water depending on demand, and up to 48% of the recirculated water did not transit through the flash water heater. The risk of *Lp* proliferation in heat exchangers is exacerbated by (1) the prevailing environmental conditions (eg, temperature, surface area, surface-to-volume ratio, materials); (2) operational conditions (eg, low flow, stagnation); and (3) the microbial load and presence of *Lp* in the feed water, which was the case in wing A for the recirculated water feed. The presence of the outbreak strain in the system for the past years combined with feeding contaminated recirculated water into a heat exchanger providing ideal growth conditions likely culminated in the higher *Lp* loads measured in wing A and a higher risk of infection. The contamination observed in wing B was associated with areas having recirculation deficiencies, preventing the hot water temperature from being maintained in these areas. Resolution of the identified deficiencies contributed to the reduction in *Lp* contamination observed.^9^

The physical characteristics and operating conditions of heat exchangers provide ideal conditions for biofilm formation and the development of opportunistic pathogens. In the present study, although physical characteristics of the heat exchangers were similar in the 2 wings, the piping diagram and operating conditions were different. Design and operation of hot water system should prevent *Lp* proliferation and prevent the conditions in which amoebae-hosting biofilms develop. Furthermore, hot water system operators should not rely on the passage of water through the water heater to inactivate *Lp* from the recirculating stream and makeup preheated water. Short exposure to elevated temperatures may not be sufficient to inactivate certain strains of *Lp* shown to resist high temperatures (70°C) for prolonged periods (60 minutes) and to develop heat resistance after shock treatment.^25^ ^,^ ^26^

Our study was subjected to a number of limitations. First, although the same strain was isolated in the system in 1995, we could only hypothesize that it was present continuously in the system over the 20 years preceding the outbreak in 2014 since there was no monitoring of *Lp* performed in the hot water system during those years. Second, PFGE could not be performed on strains isolated from the hot water system samples 1 year after the outbreak. Third, owing to the urgency to apply corrective measures, the observed decrease in system contamination cannot be attributed to a specific corrective measure but rather to the sum of actions that were taken to eradicate the contamination. Finally, sampling of the heat exchangers was not performed at the time of the outbreak because they were not suspected initially.

In healthcare facilities serving patients more vulnerable to legionellosis, the risk associated with the installation of such devices needs to be carefully evaluated with respect to the important costs associated with legionellosis hospitalization (US $34,000/episode) and the elevated mortality rate.^1^ Estimated energy savings in the present case study ranged from US $700 to US $1,700 per month. The addition of energy conservation devices and operational procedures should be evaluated by the water safety committee together with the infection prevention and control team, and weighed against the risk of exposing patients and the burden of preventive monitoring.
